# Enterocyte Autoantibodies (GECAs) and HLA: Their Relationship with HIV Infection Pathogenesis

**DOI:** 10.3390/ijms27031254

**Published:** 2026-01-27

**Authors:** Antonio Arnaiz-Villena, Tomas Lledo, Christian Vaquero-Yuste, Ignacio Juarez, Jose Manuel Martin-Villa

**Affiliations:** 1 Department of Immunology, School of Medicine, University Complutense of Madrid, 28040 Madrid, Spain; 2 Instituto de Investigación Sanitaria Gregorio Marañón, 28007 Madrid, Spain

**Keywords:** enterocytes, autoantiobodies against enterocytes (GECAs, gut epithelial cells autoantibodies), HLA (human major histocompatibility complex), HIV, AIDS, immune deficiency, autoimmunity, microgenobioma, pathogenesis, diagnosis, retrovirus

## Abstract

The significance of gut epithelial cell autoantibodies (GECAs), human leukocyte antigen (HLA) alleles, and other scientifically relevant factors has been largely overlooked, despite their potential importance in the medical management of HIV-infected individuals, in understanding the pathogenesis of AIDS, and in improving epidemiological and diagnostic approaches. This review may be considered as a hypothesis-driven narrative paper mostly considering GECAs and some easily detectable genetic markers. Thus, the aim is to highlight these neglected medical and scientific issues. Addressing them may contribute to a deeper understanding of HIV pathology at both the individual and population levels. Autoantibodies against enterocytes (GECAs) are present in the majority of HIV-positive patients. These intestinal epithelial cells are crucial for nutrient absorption and because of their role as antigen-presenting cells (APCs) within the immune system. Furthermore, the number of CD4-positive lymphocytes depends largely on daily antigenic stimulation rather than on thymic function, which becomes residual or inactive after puberty. The fall of CD4+ lymphocyte counts observed in HIV-infected patients may therefore be exacerbated by enterocyte dysfunction/damage, as indicated by the presence of GECAs. These autoantibodies either cause or reflect damage to these important antigen-presenting cells, which may impair intestinal antigen presentation by their surface HLA proteins to the clonotypic T-cell receptor of lymphocytes. Additionally, the association between specific HLA alleles and a CCR5 variant affects HIV disease progression or transmission and should be considered in both adults and mother–infant pairs. In particular, HLA-B35 and HLA-B57 allelic groups have been implicated in influencing both the transmission and progression of HIV infection. Moreover, several aspects of the natural history of HIV infection remain unresolved and controversial, and these issues warrant urgent clarification. For instance, diagnostic tests are not yet standardised globally, and viral abundance in HIV-infected individuals or AIDS patients’ cells may be relatively low. In summary, the neglected facets of HIV infection demand renewed investigation, particularly now that an HIV diagnosis is no longer the devastating prognosis it once was. The objective of this work is to emphasise additional factors that may influence the course of AIDS, such as enterocyte injury reflected by presence of GECAs. Ultimately, we propose that GECAs may impair enterocytes’ HLA (MHC II)-mediated antigen presentation by enterocytes to CD4+ T lymphocytes (through T-cell receptors), thereby diminishing T-cell proliferation, reducing CD4+ cell numbers, and impairing immune function.

## 1. Introduction and Physiopatology

**Figure 1:**
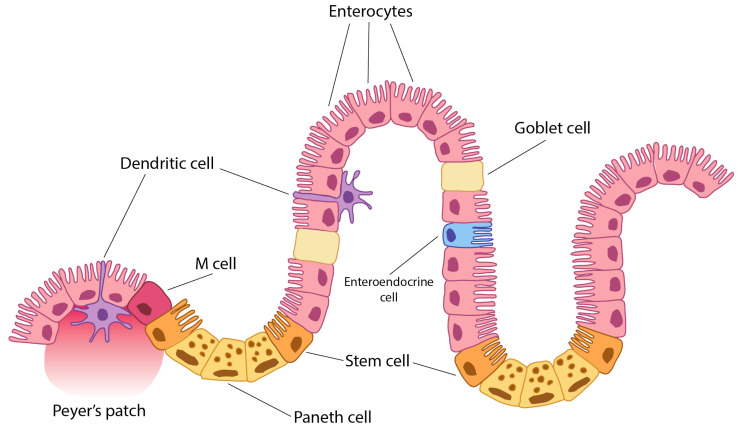
Peyer’s patch is a lymphoid tissue cell accumulation in mucosa and submucosa of the ileum (small intestine). A dendritic cell is an immune tissue-presenting cell. An M-cell (microfold cell) is a cell which may have been originated from dendritic cells. A Paneth cell is a specialised epithelial cell which secretes defences and lysozyme and other antimicrobials. It also serves as a growth factor and casts signals to stem cells to regenerate intestinal epithelium. They originate from stem cells and have control in microbiota and intestinal immunity. Stem cells are also known as Lgr5+ cells. Enterocytes are the most numerous cells along the small intestine (duodenum, jejunum, and ileum). However, they are also present in the large intestine (colon and appendix). They are absorptive and antigen-presenting cells. All of these cells, and particularly enterocytes, are important for antigen tolerance of immune response at the intestine level by promoting either tolerance or immune response. A goblet cell is a specialised epithelial cell which secretes mucus [7].

The environment of the intestinal immune system may be one of the most complex in the organism [1]. To discriminate between harmful or harmless antigens and commensal and non-commensal bacteria is one of the challenges of the intestinal immune system [1,2,3]. Different cells of the intestine are also continuously sampling and screening this huge number of antigens. In the intestinal epithelium, there are epithelial and immune cells which are continuously sampling the intestinal content. The predominant epithelial cells are enterocytes (Figure 1) [1,2,3,4], and M-cells and dendritic cells are the professional antigen-presenting immune cells. In addition to their nutrient absorptive function, epithelial cells of the small intestine (enterocytes) can sample and screen antigens in the intestinal lumen [1,2,3,4,5,6]. Moreover, these enterocytes may present antigens to lymphocytes through class II MHC during either inflammation or normal conditions [1,2,3,4]. Thus, enterocytes are important cells in mucosal infections and in future oral vaccination.

Thus, the aim of this short review is to summarise the effect of specific autoantibodies on enterocytes (gut epithelial cell autoantibodies, GECAs), their triggering by HIV, and also to provide an overview of aspects of the intestinal immune system related to the antigen-presenting function of enterocytes and its implications for intestinal and general HIV pathogenesis, since antigens and cytokine signals act on stem cells to regenerate the intestinal epithelium. They originate from stem cells and exert control over the microbiota and intestinal immunity. Stem cells are also known as Lgr5+ cells. Enterocytes are the most numerous cells along the small intestine (duodenum, jejunum, and ileum). However, they are also present in the large intestine (colon and appendix). They are absorptive and antigen-presenting cells. All of these cells, and particularly enterocytes, are important for antigen tolerance or immune responses at the intestinal level, by promoting either tolerance or active immunity. The goblet cell is a specialised epithelial cell that secretes mucus [7]. Presentation is a main mechanism to maintain T-cell number and a numerous APC pool is constituted by enterocytes in addition to other presenting cells.

### 1.1. Gut Epithelial Cells (Enterocytes) Are Genuine Antigen-Presenting Cells

Not only M-cells (epithelial specialised intestinal cell) act like APC, but also enterocytes may present antigens to naïve or memory CD4+ T-cells. This is possible because class II MHC molecules are expressed by enterocytes during intestinal inflammation or even normal condition, although enterocytes in the colon only seem to be active in inflammatory processes [1,2,3,4,5,6]. Enterocytes can interact with intraepithelial lymphocytes (IEL) with interactions cell-to-cell but also with interactions like exosome-to-cell [8]. In addition, enterocytes can secrete at basolateral side exosome-like vesicles which are completely recovered of class II MHC molecules in inflammatory conditions [8]. These epithelial cells could activate lymphocytes perfectly in the intestinal inflammation [4]. Also, the enterocyte signal by class II MCH molecules allows T-cell proliferation. In summary, enterocyte is an APC in inflammatory conditions [4].

**Figure 2:**
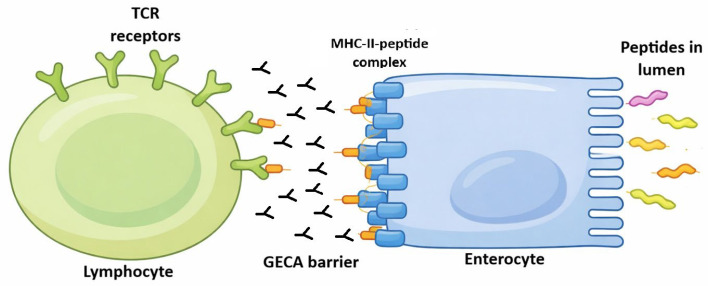
Enterocyte is an antigen-presenting cell. Different proteins, which are located in intestinal lumen, may bind to enterocyte villi. These antigens would be led to base-lateral side of enterocytes by transepithelial transcytosis. These proteins may be absorbed by enterocytes because of their absorptive function. After peptide processing by enterocytes, the peptides would be loaded in MHC class II (HLA) molecules extant on enterocyte surface in order to be presented (another presentation way may involve a specialised Ig Fc receptor). This presentation would be made by enterocyte interaction with a T lymphocyte through the lymphocyte T-cell receptor. Gut epithelial cell autoantibodies (GECAs) could hinder this presentation by damaging enterocytes at their MHC–peptide interaction or villi absorption function. The fact that only villi and adjacent tissue seem to be the only target of GECAs (Figure 3) may or may not be an artefact of the immune fluoresce technique. There is the possibility that other parts of enterocytes are also damaged by GECAs or these represent HIV-induced enterocyte damage by other unknown mechanisms. “GECA barrier” only means that the immune function of enterocyte as presenting cells may be damaged as marked by the presence of GECAs [7].

On the other hand, enterocytes have other ways to present antigens to T lymphocytes. Enterocytes express an FcR at the apical side (in the brush border) [6]. This receptor is the FcRn, neonatal FC receptor (Figure 2). In rodents, FcRn expression is limited to the neonatal time in rodents; however, in humans, FcRn is expressed throughout life [6]. The immunoglobulin–FcRn complex is expressed in the brush border of enterocyte to sample antigens in the intestinal lumen. If there is a specific antigen to bind to the Ig-FcRn complex, it goes back to the basement by transcytosis.

These dual mechanisms by which enterocytes act as antigen-presenting cells (both HLA and FcRn molecules) may also yield harmful consequences. Firstly, the activation of lymphocytes through class II MHC molecules in response to harmless antigens may contribute to diseases such as inflammatory bowel disease (hereafter ‘IBD’) [1]. If a gastrointestinal condition is not adequately resolved and inflammation persists, enterocytes might present a harmless antigen as if it were pathogenic, thereby initiating IBD. Secondly, the pathology of coeliac disease may be exacerbated by the binding of gliadin to the IgA–FcRn complex and its subsequent transepithelial uptake [6].

Thus, further investigations and deeper insights are required into the mechanisms of antigen presentation by enterocytes, the processes in which this presentation is involved, and its implications for both physiological and pathological states—particularly in conditions with pronounced intestinal tropism, such as early HIV infection or IBD.

We are going to review some aspects mainly epithelial and genetic of the HIV infection, since reviews have already been mostly focused on and dealing with leukocytes and their activity in relation with HIV. Thus, the following block of reviews are clearly related to (1) enterocyte pathology, and (2) gut epithelial cell autoantibodies and the infected individual genetics regarding CCR5 and HLA variants. The latter both take into account horizontal and vertical maternal foetal transmission.

### 1.2. HIV Infection and Intestinal Damage with the Presence of Gut Epithelial Cell Autoantibodies (GECAs)

There is evidence supporting the hypothesis that immunodeficiency in acquired immunodeficiency syndrome (AIDS) arises not solely from the direct effects of human immunodeficiency virus (HIV), but also from additional factors, such as autoimmunity (gut epithelial cell autoantibodies, GECAs) and genetic influences (human leukocyte antigen, HLA), which contribute to disease pathogenesis [9]. The intestine represents a primary target for HIV [10,11], where profound depletion of CD4+ T-cells persists despite highly active antiretroviral therapy (HAART) [12]. Loss of antigen-presenting cells (APCs) or their functionality constitutes a critical determinant of immunodeficiency in HIV-infected individuals [9,13]. In adults, peripheral T-cell homeostasis relies heavily on antigenic stimulation, with thymic contribution being negligible or absent post-puberty owing to its involution into non-functional fatty and fibrous tissue. Dendritic APCs exhibit defects in AIDS [9,13], while enterocytes sustain damage during HIV infection [14,15], accompanied by GECAs in 71% of affected patients [15]. Thus, depletion of intestinal CD4+ T-cells may stem from absent class II major histocompatibility complex (MHC) survival signals provided by enterocytes.

Several mechanisms underlie enterocyte injury: HIV impedes glucose uptake via virotoxic effects [14]; it elevates intracellular calcium, disrupting ion homeostasis [14]; and it accelerates enterocyte turnover, yielding deformed and apoptotic cells [14]. HIV infection further provokes exacerbated intestinal inflammation, compounding enterocyte damage [14]. Persistent HIV reservoirs in infected CD4+ cells perpetuate viraemia upon lysis, fostering GECAs and other autoantibodies of uncertain clinical significance [16]. Such cellular perturbations may expose enterocyte antigens, eliciting immune responses inclusive of GECAs in HIV patients [14], potentially initiating or exacerbating intestinal injury (Figure 2). GECAs prevalence reached 71% in HIV-infected individuals, contrasting starkly with its absence in healthy blood donors [15]; perinuclear anti-neutrophil cytoplasmic autoantibodies (pANCA) occurred in 25% of cases, again absent in controls [15].

This specificity argues against the attribution of hypergammaglobulinaemia or polyclonal autoimmunity, as assays for cytoplasmic ANCA, thyroglobulin, thyroid microsomal, and glomerular basement membrane antibodies revealed no elevation [15]; nevertheless, anticardiolipin and anti-nuclear antibodies have been documented in HIV cohorts [16]. Adult CD4+ T-cell maintenance depends profoundly on antigenic drive; GECAs and enterocyte loss—representing a major APC population—disrupt this, yielding CD4+ attrition beyond direct viral cytopathicity [9,13]. Compromised enterocyte presentation impairs T-cell expansion and function [16], with IgG2 deficiency noted in patients bearing apical GECAs [17] and diminished mitogen-induced T-cell proliferation [17]. Thus, enterocyte attrition precipitates CD4+ T-cell numerical decline, proliferative failure, and functional impairment [17].

**Figure 3:**
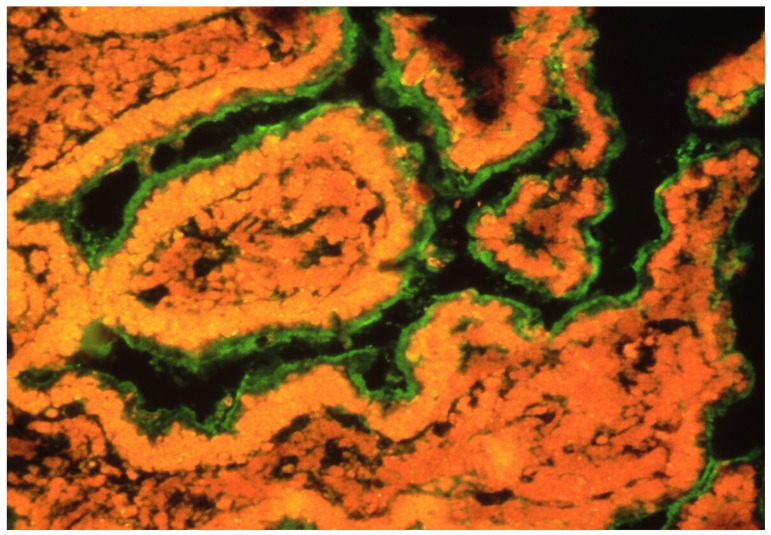
Presence of GECAs in an intestine in an HIV-infected patient with enterocyte damage [17]. GECAs (green) were detected by serum incubation of intestinal fresh normal tissue and subsequent staining by anti-Igs fluorescent MoAb.

These findings on HIV infection effects in the intestine supports a larger role for mucosal sites (mainly the intestine) in HIV replication and AIDS pathology [12]. Moreover, all these data suggest that our understanding of HIV infection—especially knowledge dedicated to vaccine design and therapeutic strategies—has been biased by focusing solely on peripheral blood examinations [12]. Studies based on peripheral blood provide only a limited view of AIDS pathogenesis [12]. This perspective implies that it would be erroneous to aim therapies only at correcting T-cell function or preventing infection of T-cells.

### 1.3. CCR5 Variant and RANTES During HIV Infection in the Small Intestine Enterocytes

Regarding *genetic* susceptibility factors, a CCR5 HIV co-receptor variant, CCR5 Δ32, apparently protects against HIV infection progression, which is another important genetic factor together with some HLA alleles. It has been described that CCR5 is a major co-receptor for infection in R5-tropic HIV strains [18,19,20,21,22], but the CCR5 Δ32 polymorphism confers resistance to this infection [22,23], notably among men who remained uninfected despite repeated high-risk exposure. Individuals homozygous for this polymorphism (Δ32/Δ32) exhibit full resistance to R5-HIV infection and replication [23,24,25,26,27]. Not only do these immune characteristics support resistance to infection progression, but high levels of the chemokine RANTES (CCL5) also contribute to protection [22]. Thus, both elevated RANTES levels and low CCR5 expression may confer resistance against R5-HIV infection [25,28,29]. It is noteworthy that RANTES may be released by enterocytes via cytokine induction during infection [30,31,32,33]. Additionally, cytokine induction in enterocytes may provoke the expression of CCR5 and CXCR4 (the co-receptor for X4-tropic HIV strains) [30]. Under infection and inflammatory conditions, CCR5, CXCR4, and RANTES are likely involved in regulating mucosal immunity in the intestine, inhibiting HIV transmission and infection between enterocytes and lymphocytes. In summary, the main relationship between CCR5 and RANTES during HIV infection is that signalling mediated by these molecules regulates the recruitment and activation of immune cells to the intestinal epithelium, where enterocytes participate crucially in antigen presentation under infectious or inflammatory conditions.

### 1.4. HLA Immunogenetics and AIDS

Genetic factors also influence HIV disease progression. Clinical variability among patients infected with HIV is linked to different genetic backgrounds. Inherited factors contribute to resistance against HIV in exposed individuals, modulate the rate of disease progression, and affect transmission likelihood. Among the genetic variants impacting the course of HIV infection, human leukocyte antigen (HLA) class I genes show the strongest and most consistent association, underscoring the critical role of CD8+ T-cells in viral control. HLA proteins are essential in T-cell-mediated adaptive immunity by presenting dominant HIV epitomes to cytotoxic T lymphocytes (CTLs) and CD4+ T-cells. Furthermore, genetic and functional studies suggest that HLA contributes to natural killer-cell-mediated innate immunity against HIV through interactions with killer-cell immunoglobulin-like receptors (KIR). The CCR5 receptor polymorphism is also significant in determining infection susceptibility. Many of these genmarkers relate to immune response modulation, such as HLA [34]. Certain HLA genes exhibit some of the strongest and most consistent associations with HIV disease progression [34]; HLA proteins play vital roles in adaptive immunity by presenting immunodominant HIV epitopes to CD8+ CTLs and CD4+ T-cells [34]. Thus, HLA markers/alleles are useful for HIV infection clinical management, particularly for prognosis and treatment. See Table S1 in the Supplementary Materials, where details of each HLA marker effect on the HIV infection course are shown [35,36,37,38,39,40,41,42,43,44,45,46,47,48,49,50,51,52,53,54,55,56,57,58,59,60,61,62,63,64,65,66,67,68,69,70]. HLA-B*57 is important in HIV mainly as a prognostic marker of slower disease progression and as a safety marker for abacavir hypersensitivity. It also has implications for long-term outcomes and for selecting or avoiding specific antiretroviral drugs. HLA-B57, especially B57:01, occurs in a small fraction of the general population but is markedly enriched among HIV “elite controllers” or long-term non-progressors who maintain low viral loads without therapy. This allele is associated with stronger CD8+ T-cell responses against conserved HIV viral Gag epitopes and a lower viral set point, which correlate with slower CD4 decline and delayed progression to AIDS in many, though not all, carriers. HLA-B*57:01, *57:02, and *57:03 are closely related but differ in how strongly they protect against HIV disease progression and in their relevance for drug hypersensitivity testing. HLA-B*57:01 is the most clearly “protective” allotype, strongly enriched among elite controllers and associated with slower progression and better viral control in many cohorts. HLA-B*57:03 is also associated with better HIV control, especially in African populations, but generally shows a somewhat weaker protective effect than *57:01. HLA-B*57:02 is less well studied; available data suggest that it is not as protective as *57:01 and *57:03 and may be associated with higher viral loads than *57:03 in some African cohorts. Carriers of *57:01 or *57:03 more often have lower viral set points and slower CD4 decline, although not all individuals are protected and some progress normally.

On the other hand, HLA-B*35 is not used to diagnose HIV; diagnosis relies on serologic/antigen–antibody tests and HIV RNA tests. Prognostic and monitoring tools remain CD4 count, HIV viral load, and clinical staging; HLA typing (including B*35) is mainly a research or specialised prognostic marker, and is not routine in clinical care. Some cohort data suggest HLA-B*35 is associated with poorer immune reconstitution or higher risk of “nonresponse” (suboptimal CD4 recovery) despite virologic suppression, which may justify closer monitoring and aggressive management of comorbidities in carriers. In summary for a patient with HIV who is HLA-B*35 positive, key implications are a higher baseline risk of rapid progression, so early diagnosis and immediate initiation of treatment is particularly important.

### 1.5. HIV, HLA, and Mother-to-Child Transmission

It is noteworthy that HLA-B35 may influence vertical transmission of HIV from mother to child [71]. No differences were observed in the distribution of this HLA allele between transmitting and non-transmitting mothers; however, the HLA-B35 allele was more frequent among infected children than among their non-infected counterparts [71]. Evidence from prior studies indicates that HLA-B35, Cw4, and DR4 alleles elevate the risk of infection and progression to AIDS following vertical HIV transmission, underscoring the relevance of inheritance patterns and suggesting that HLA genetics exert a significant yet complex influence on transmission and disease progression in children [72].

A subsequent systematic review demonstrated that HLA-B polymorphisms substantially affect both vertical HIV-1 transmission and disease progression in children [73]. Certain alleles confer protection (B57, B81, and B53:01), whereas others—particularly within the B35 group—are linked to heightened infection risk and rapid AIDS progression. Larger studies are nonetheless required to confirm these findings, given that over 50% were conducted in Africa, where population size and genetic variability constrain generalisation [74,75]. Diagnostic test standardisation also varies across countries, potentially yielding divergent results. The human genome composition, moreover, has been overlooked for diagnostic anomalies in diagnostic interpretation. It harbours at least 8% retroviral sequences [76,77,78,79,80,81,82] and test variations should be homogenised around the world.

## 2. Conclusions

1. GECAs are anti-enterocyte autoantibodies that appear to attack, or reflect damage to, one of the largest populations of antigen-presenting cells (APCs), the enterocytes, which are epithelial cells in the small intestine and colon.This damage in such a big number of APCs is beginning to be more apparent with time inovert AIDS patients.
2. In the interaction among HIV, co-receptors on lymphocytes like CCR5 variants, antigen-presenting enterocytes molecules (HLA), production of the lymphokine RANTES, and CD4+ cells (clonotypic T-cell receptor)are fundamental for the different course of HIV infection in different patients, alongside other inherited genetics non clearly established molecules.
3. Genetic factors clearly linked to disease progression warrant further study, focusing not only on HLA class I and class II antigens but also on other HLA loci variants like non-classical HLA-E,-F, -G allelesand killer-cell immunoglobulin-like receptors (KIR).
4. The numbers of CD4+ lymphocytes in adults greatly depend on the abundance of healthy and functional antigen-presenting cells such as enterocytes, since the thymus is non-functional in adult humans. Thus, damage of enterocytes will result in a defective antigen presentation at the local level and an impaired immunity.
5. The epithelium is a neglected subject in both immunology textbooks and research projects on immune disease pathogenesis including HIV infection. Therefore, it may be fruitful to study enterocytes more extensively in the context of HIV infection, with simultaneous efforts toward vaccine development.
6. In summary, the main message of this paper is that GECAs may harm enterocytes (or reflect it), a numerous and major type of antigen-presenting cells (APCs). This harm may reduce the antigenic stimulus necessary to maintain CD4+ T-cell population numbers. Coupled with possible GECA targets such as HLA class II molecules on enterocytes and co-stimulatory pathways critical for T-cell activation and proliferation, these effects may explain a relationship between GECAs and the interaction of the T-cell receptor (TCR), HLA, and CD4 molecules, leading to a reduction in CD4+ cells and defective antigen presentation within the immune system.Thus, it may finally help to HIV infection final stage of AIDs, which is rarely seen today in our hospitals.

## Data Availability

No new data were created or analyzed in this study. Data sharing is not applicable to this article.

## Supplementary Materials

The following supporting information can be downloaded at https://www.mdpi.com/article/10.3390/ijms27031254/s1.
