# Supplementary material for: Enterocyte Autoantibodies (GECAs) and HLA: Their Relationship with HIV Infection Pathogenesis
Source: Int J Mol Sci. 2026 Jan 27;27(3):1254. doi: 10.3390/ijms27031254 (PMC12898150; doi:10.3390/ijms27031254)
Supplement: Supplementary file 1 [file ijms-27-01254-s001.zip › ijms-3862481-supplementary.docx]

**Supplementary material 1**

Effects in progression to AIDS from HIV infection by different HLA markers. See also [32].

| ***HLA* genetic marker** | **Effect on HIV infection** | **Cohort ethnicity** | **Reference** |
| --- | --- | --- | --- |
| ***A*02:01*** | Susceptibility to infection | Spaniards | [34] |
| ***A*03*** | Favourable for LTNP condition | Spaniards | [35] [36] |
| ***A*24*** | Unfavourable for LTNP condition | Spaniards | [35] |
| ***A*25/25:01*** | Slow disease progression | Caucasian | [37] [38] |
| ***A*29*** | Accelerated disease progression (LTNP) | Spaniards | [35] |
| ***A*32/32:01*** | Slow disease progression | Caucasians  African Americans from the US | [37] [39]  [40] |
| ***A*36:01*** | Accelerated disease progression | Africans (South Africa, Zambia, Zimbabwe, Bostwana) | [41] [42] |
| ***A*74/74:01*** | Slow disease progression | Africans (South Africa, Tanzania, Zambia, Zimbabwe, Bostwana)  African Americans | [41] [43] [40] [44] |
| ***B*07:02*** | Accelerated disease progression | Caucasians | [38] |
| ***B*08/08:01*** | Accelerated disease progression (LTNP) | Caucasians | [38] [35] |
| ***B*13/13:02*** | Slow disease progression | South Africans  Caucasians | [39] [38] |
| ***B*14/14:02*** | Slow disease progression | Caucasians  African Americans | [38] [40] |
| ***B*18*** | Risk allele for HIV infection Accelerated disease progression | Caucasians | [45]  [46] [42] |
|  | Favourable for LTNP condition Protection against infection | Spaniards  Indians | [47] [48] |
| ***B*27/27:05*** | Slow disease progression Lower viremia Longer survival | Caucasians | [37] [49] [50] [39] [38] [36] |
| ***B*35/35:01*** | Accelerated disease progression | Caucasians  North American Europeans | [51]  [49] [52] |
| ***B*35:02/35:03*** | Accelerated disease progression | Caucasians | [52] [38] |
| ***B*39*** | Slow disease progression (LTNP) | Spaniards | [35] |
|  | Accelerated disease progression | Argentinians  Indians | [45]  [48] |
| ***B*42/42:01*** | Slow disease progression | Africans (South Africa, Bostwana, Zimbabwe) | [42] |
| ***B*44:03*** | Slow disease progression | Africans (South Africa, Bostwana, Zimbabwe) | [46] [42] |
| ***B*45/45:01*** | Accelerated disease progression | Africans (South Africa, Zambia, Zimbabwe, Bostwana) | [41] [42] |
| ***B*51/51*01*** | Slow disease progression | Caucasian  Africans (South Africans, Bostwanans, Zimbabweans) | [37] [49] [42] |
| ***B*52/52:01*** | Slow disease progression | Caucasians  Brazilians | [35] [38] [53] |
| ***B*53:01*** | Accelerated disease progression | Caucasians  African Americans | [54] [38] |
| ***B*54*** | Accelerated disease progression | Caucasians | [55]  [56] |
| ***B*55*** | Accelerated disease progression | Caucasians | [55] [56] |
| ***B*56*** | Accelerated disease progression | Caucasians | [55] [56] |
| ***B*57/B*57:01*** | Slow disease progression Lower viremia Longer survival | Caucasians | [57]  [51] |
| ***B*57:02*** | Slow disease progression | South Africans | [46] |
| ***B*57:03*** | Slow disease progression | Africans (Rwandans, Zambians, South Africans, Zimbabweans, Bostwanans)  African Americans | [58]  [46] [41] [38] [40] [42] |
| ***B*58:01*** | Slow disease progression | Africans (Rwandans, Zambians, South Africans, Zimbabweans, Bostwanans) | [59] [42] |
| ***B*58:02*** | Accelerated disease progression | Africans (South Africa, Tanzania, Zambia, Zimbabwe, Bostwana) | [59] [41] [42] |
| ***B*81:01*** | Slow disease progression | Africans (South Africa, Tanzania, Zambia, Zimbabwe, Bostwana) | [59]  [41] [61] |
| ***Bw4/Bw4*** | Control of the viremia and slow disease progression | Caucasians | [60] |
| ***Bw6/Bw6*** | Accelerated disease progression | Caucasians | [35] |
| ***C*  rs9264942 (SNP)*** | Lower viral load  Slow progression to AIDS | Unknown | [62] |
| ***Cw4*** | Accelerated disease progression | Caucasians | [51] |
| ***DRB1*01*** | Protection against infection | Africans (Kenians and Bostwanans) | [63] [64] |
| ***DR13 supertype*** | Slow disease progression | Unknown | [65] |
| ***DRB1*13-DQB1*06 haplotype*** | Slow disease progression | Unknown | [65] |
| ***DRB1*13:01*** | Favourable for LTNP condition | Mixed (mostly Africans, South Americans and Caucasians) | [66] |
| ***DRB1*13:02*** | Favourable for LTNP condition | Mixed (mostly Africans, South Americans and Caucasians) | [66] |
| ***DRB1*13:03*** | Reduced viral load levels Favourable for LTNP condition | Mixed (mostly Africans, South Americans and Caucasians)  Predominantly South Africans | [66]  [67] |
| ***DRB1*13:10*** | Favourable for LTNP condition | Mixed (mostly Africans, South Americans and Caucasians) | [66] |
| ***DRB1*15:03*** | Increased susceptibility to infection | Africans (Kenians and Bostwanans) | [63] [64] |
| ***DQB1*03:02*** | Protection against infection | Spaniards | [34] |
|  | Accelerated disease progression | Caucasians | [68] |
| ***DQB1*06:02*** | Susceptibility to infection | Caucasians | [69] |
| ***DQB1*06:03*** | Protection against infection | Caucasians | [69] |
| ***DQB1*06:05*** | Susceptibility to infection | African Americans | [69] |

Table S1. depicts some HLA markers which have been described as being important in the progression to AIDS [32].
